# Developing a Novel and Convenient Model for Investigating Sweat Gland Morphogenesis from Epidermal Stem Cells

**DOI:** 10.1155/2019/4254759

**Published:** 2019-02-04

**Authors:** Tian Hu, Yongde Xu, Bin Yao, Xiaobing Fu, Sha Huang

**Affiliations:** ^1^School of Medicine, Nankai University, Tianjin 300052, China; ^2^Wound Healing and Cell Biology Laboratory, Institute of Basic Medical Sciences, General Hospital of PLA, Beijing 100853, China; ^3^Department of Urology, Beijing Friendship Hospital, The Capital University of Medical Sciences, Beijing 100050, China; ^4^Key Laboratory of Tissue Repair and Regeneration of PLA, Beijing Key Research Laboratory of Skin Injury, Repair and Regeneration, First Hospital Affiliated to General Hospital of PLA, Beijing 100048, China

## Abstract

Sweat glands developed from the embryonic epidermis. To elucidate the underlying mechanisms of morphogenesis, a reliable in vitro test system for bioactive screening must be developed. Here, we described a novel and convenient model by coculturing embryonic tissue and epidermal stem cells (ESCs) using Transwell insert for evaluating the effects of soluble morphogens on sweat gland morphogenesis in vitro. Using this coculture system, morphological alteration, histological features, and specific markers were observed. Initial experiments revealed that ESCs cocultured with embryonic paw pad (EPP) tissue demonstrated glandular structure and cytokeratin 8 (K8) and cytokeratin 18 (K18) positive, while ESCs cocultured with embryonic dorsal skin demonstrated “sea snail” structure and K8, K18 negative. Moreover, bone morphogenetic protein 4 (BMP4) and epidermal growth factor (EGF) concentrations were detected in the medium of the EPP group. BMP receptor inhibitor could effectively block the ESC differentiation to sweat glands, while EGF receptor blocker did not show the effect. Our results showed clear benefits of this novel and convenient model in terms of in vitro-in vivo correlation. It was an appropriate alternative for screening of potential bioactives regulating the sweat gland morphogenesis mechanism.

## 1. Introduction

As external temperature is not lower than the body temperature, sweat vaporization becomes the main channel for heat radiation [1]. Moreover, sweat glands contribute to skin homeostasis and involved in wound healing of the human skin. However, this gland is not fully characterized as lacking the appropriate research models. Sweat glands are only located in some distinct regions of certain mammals [2]. In comparison with other skin appendages, essential morphogens and bioactives in the process of sweat gland development are far from clear. An in vitro test system of sweat gland development for further investigation is necessary.

Since sweat glands are originated from epidermal stem cells at embryonic stage, epidermal stem cells (ESCs) were regarded as ideal seed cells for sweat gland regeneration. Our previous study found that ESCs could be induced into sweat gland cells when they were cultured in 3D condition with paw pad homogenate of mice and epidermal growth factor (EGF) [3]. Meanwhile, sweat gland function was partially recovered after transplanting tissue engineering skin with the sweat gland cells into a mouse paw pad scalded model [3]. Sweat gland niches, or specific local microenvironment, are composed of surrounding cells and extracellular matrix in the integumentary system. Secreted soluble factors, adhesion proteins, and glycosaminoglycan are some of irreplaceable components in the extracellular matrix. Studies have demonstrated that cellular niches are playing dominating roles in numerous aspects of cell behavior, for instance, cell distribution and cellular migration and differentiation [4]. In sweat gland developmental niches, soluble factors, a group of proteins secreted by basal or surrounding cells, involve in cellular differentiation, metabolism, and proliferation with extensive bioactivities. The increase of EGF and bone morphogenetic protein (BMP) was reported and detected in the extracellular matrix of epithelial-mesenchymal placodes and developing buds of sweat gland morphogenesis [5–9]. However, the bottleneck is to explain the role of these bioactives.

In this study, we aimed to mimic the physiological development of sweat glands with 3D culture. EPP tissue was incorporated into a flat-bottom culture plate, and it acted as a “mini factory” with consistent release of soluble factors into a medium. Embryonic tissue has provided a physiological microenvironment for the test system. Furthermore, we demonstrate differences in ESC differentiation after the inhibition with a BMP receptor blocker in a 3D model. Thus, this novel and convenient model also would be an appropriate alternative for investigating the soluble factors in sweat gland development.

## 2. Materials and Methods

All animal procedures were approved with the guidelines of the Institutional Animal Care and Use Committee of Chinese PLA General Hospital (Beijing, China). All experiment procedures were repeated for three times.

### 2.1. Animals

Mice in a BALB/c genetic background were used for the study. Male and female mice were put together at night and separated in the next morning. Females were observed in the morning for the formation of copulatory plug and then housed with other pregnant female mice. This time point was counted as 0.5 d. Embryonic 15.5 d (E15.5), embryonic 16.5 d (E16.5), embryonic 17.5 d (E17.5), and embryonic 18.5 d (E18.5) mice were picked up for experiments.

### 2.2. Embryonic Tissue Isolation

Pregnant mice at embryonic days of 15.5, 16.5, 17.5, and 18.5 were killed and put in 75% ethanol (Beijing Chemical Works, Beijing, China) for 15 min; fetal mice were then removed from the uteri. EPP tissue and dorsal skin were collected from the fetuses using a dissecting microscope under sterile conditions. Embryonic tissue was cut into tiny pieces and weighed on an electronic scale (JM-B 2003, Zhuji, Zhejiang, China) in a sterilized condition; 10 mg of tissue was added in a well.

### 2.3. Epidermal Stem Cell Isolation

The dorsal skin down was cut from fetal mice at embryonic day of 15.5 and diced to pieces about 0.5 cm × 0.5 cm. The pieces were digested in 2 mg/ml dispase overnight at 4°C, then the epidermis and dermis were separated with tweezers, and the dermis was discarded. Then, the epidermis was minced and placed in 0.25% trypsin-EDTA for 20 min at 37°C. All the suspension passed the cell strainer (431752, Corning, NY, USA) and dropped in a 50 ml centrifuge tube to centrifuge at 1000 rpm for 5 min. The pellet is ESCs and resuspends with DMEM/F-12 (11320033, Gibco, Waltham, MA, USA) and seeded on 100 mm culture dishes. All operations were under sterile conditions.

### 2.4. Establishment of the Development Model

**Figure 1:**
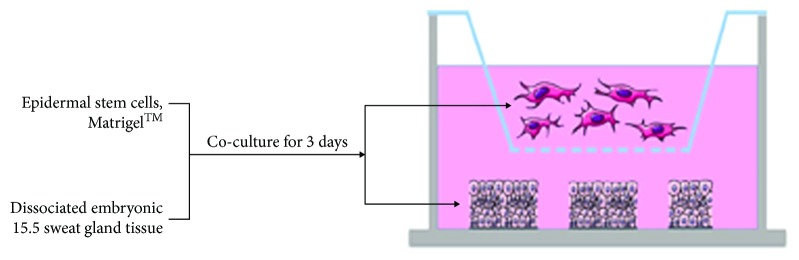
Schematic representation of the methods used for in vitro induction of sweat gland development by 3D culture epidermal stem cells and embryonic tissue. Embryonic tissue was incorporated into the 24-well flat-bottom plates as the developmental environment. Epidermal stem cells and Matrigel™ were cultured in Transwell culture plates.

Embryonic tissue contained in 1 ml of DMEM/F12 medium with 10% FBS was set per well of a 24-well flat-bottom culture plate. A Transwell cell culture insert with a pore size of 0.4 *μ*m (3413, Corning, NY, USA) was placed in each well containing 1 ml of 2.5 × 10^5^ ESCs in DMEM/F12 medium with 50% Matrigel working solution (Corning, 354230, NY, USA). All procedures with Matrigel were performed on ice. The schematic illustration of inducing systems is represented in Figure 1.

### 2.5. Immunofluorescence Staining and H&E Staining of Cell Clusters and Tissue

For immunofluorescence, cell clusters or tissues were fixed with 4% paraformaldehyde for 10 min and permeabilized with 0.2% Triton X-100 in PBS for 20 min. Cell clusters or tissues were blocked with a blocking buffer (10% goat serum, 1% BSA, 0.2% Triton X-100, and PBS 1X) for 1 h and then stained with primary antibodies: mouse monoclonal anti-K18 antibody (ab668; Abcam, Cambridge, MA, USA), rabbit monoclonal anti-K8 antibody (ab53280; Abcam, Cambridge, MA, USA), rabbit monoclonal anti-K14 antibody (ab181595; Abcam, Cambridge, MA, USA), and rabbit monoclonal anti-K5 antibody (ab52635; Abcam, Cambridge, MA, USA) overnight at 4°C. After that, cells were incubated with the secondary antibody goat anti-mouse-IgG-Alexa Fluor 488 antibody (ab150117; Abcam, Cambridge, MA, USA), goat anti-rabbit-IgG-Alexa Fluor 488 antibody (ab150077; Abcam, Cambridge, MA, USA), goat anti-rabbit-IgG-Alexa Fluor 594 antibody (ab150080; Abcam, Cambridge, MA, USA), and goat anti-mouse-IgG-Alexa Fluor 594 antibody (ab150116; Abcam, Cambridge, MA, USA) for 30 min. The nuclei were stained with DAPI for 25 min. Cells were observed using a fluorescence microscope Olympus BX51, and images were acquired with a digital camera (Camedia C4040, Olympus, Tokyo, Japan).

For histological analysis, cell clusters and embryonic tissue were fixed in 3% paraformaldehyde for 24 h and embedded in OCT or paraffin. Seven-micron-thick sections were stained with hematoxylin and eosin (H&E staining) according to routine histological protocols.

### 2.6. Enzyme-Linked Immunosorbent Assay

Supernatants were collected from the upper chamber of the Transwell plates at different time. BMP4 and EGF concentrations in supernatant were measured using a mouse BMP4 (CSB-E04512m, Cusabio, Wuhan, Hubei, China) and EGF ELISA kit (EM014-96, ExCell Bio, Shanghai, China). Plates were read using the Bio­Rad microplate reader at 450 nm; BMP4 and EGF concentrations were calculated from the standard curve by the plate reader software.

### 2.7. BMP Receptor Inhibitor and EGF Receptor Inhibitor

BMP receptor inhibitor ML347 (S7148, Selleckchem, Houston, TX, USA) and EGF receptor inhibitor OSL-744 (S1023, Selleckchem, Houston, TX, USA) were used in the experiment. ML347 working solution was prepared in DMEM/F12 medium with 10% FBS at a concentration of 32 nM. OSL-744 working solution was prepared in DMEM/F12 medium with 10% FBS at a concentration of 2 nM. In the development model, the working solution of both inhibitors was changed every day during culture.

### 2.8. Statistical Analysis

Statistical significance was determined by Student's *t*­test for comparisons between two groups as appropriate. A *p* value < 0.05 was considered significant.

## 3. Results

### 3.1. Comparison of Sweat Gland Development between In Vivo and In Vitro Systems

**Figure 2:**
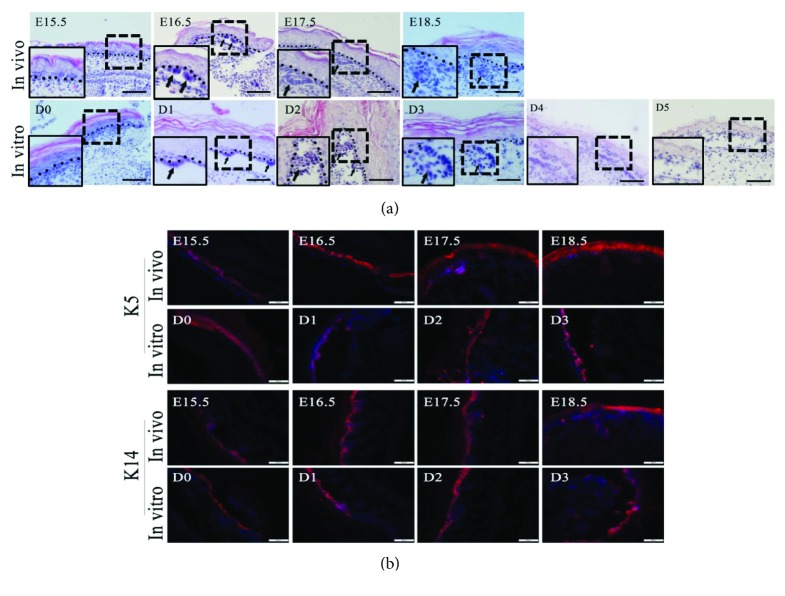
Comparison of sweat gland development between in vivo and in vitro. Sweat glands demonstrated similar developmental characteristics in vitro with those in vivo. (a) Comparison of sweat gland morphogenesis between in vivo and in vitro by H&E staining. Square with dotted line showed the region of sweat gland. Square with line in the left lower part of each picture was the enlargement of dotted square. Irregular histological structure was observed in tissue culture at days 4 and 5 (bars = 200 *μ*m). (b) Immunofluorescent staining of K5 and K14 in tissue culture (in vitro) and comparison with embryonic tissue (in vivo) at corresponding time points. All the nuclei were counterstained with DAPI (DAPI: blue; K5, K14: red) (bars = 50 *μ*m). (K5: cytokeratin 5, K14: cytokeratin 14).

To identify the availability and feasibility of the in vitro system, we compared morphological and protein expression changes between in vivo embryonic tissue and the in vitro systems. At embryonic stage of 16.5 day, placode was formed with dermal condensate in EPP tissue in BALB/c mice. At day 17.5, sweat gland bud was observed in the basal layer of the epidermis. Typical ductal structure was formed at embryonic day of 18.5 (Figure 2(a)). When EPP tissue was cultured in vitro, placode structure was formed at day 1, sweat gland bud was observed at day 2, and ductal structure was formed at day 3 (Figure 2(a)). It implied that the histological features of sweat gland development in vitro were consistent with those in vivo. However, histological structure of the epidermis and underlying dermis of EPP tissue became irregular at days 4 and 5 (Figure 2(a)). K14 and K5 are the specific keratin proteins expressed in basal epidermal cells; keratin 14 dimerizes with keratin 5 and forms the intermediate filaments which constitute the cytoskeleton of basal epithelial cells [2]. Immunofluorescence staining showed K14 and K5 expressions in the epidermis of both groups. Besides, the placode, bud, and ductal structure of both groups showed K14 and K5 expressions. It indicated that in vitro sweat gland expressed similar specific markers with those in vivo (Figure 2(b)).

### 3.2. Glandular Cell Clusters or Nonglandular Cell Clusters Were Formed from ESCs in a Different Development Model

**Figure 3:**
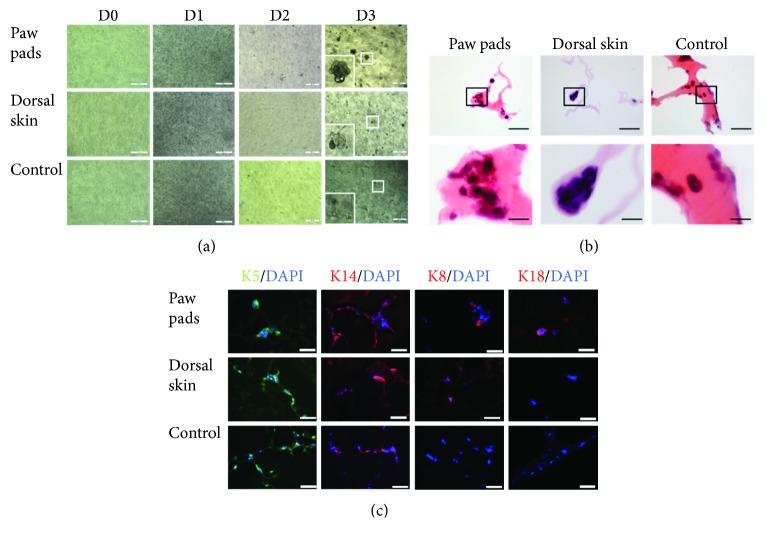
Epidermal stem cells developed into glandular cell clusters by coculture with EPP tissue. Epidermal stem cells were induced into glandular cell clusters by coculture with EPP tissue. Nonglandular or “sea snail” cell clusters were formed in the group with embryonic dorsal skin. (a) Morphological alterations of epidermal stem cells in different inducing development systems were observed by inverted microscope (all bars = 200 *μ*m). (b) Histological features of multicellular clusters were evaluated by H&E staining in different inducing development systems (bars = 200 *μ*m and 50 *μ*m). (c) Specific biomarkers of sweat gland development were detected by immunofluorescent staining in different inducing development systems. All the nuclei were counterstained with DAPI (DAPI: blue; K5: green; and K14, K8, and K18: red) (bars = 50 *μ*m). (K5: cytokeratin 5, K14: cytokeratin 14, K8: cytokeratin 8, and K18: cytokeratin 18).

To validate the effect of the coculture model, cell clusters in Matrigel were detected and compared in morphology and protein expression. In the EPP group, EPP tissue was incorporated into the 24-well flat-bottom culture plate; glandular cell clusters were formed in the upper chamber. In the blank control group, no tissue was put into a flat-bottom culture plate; branching structure with filament was formed in the upper chamber. In the dorsal skin group, ESCs formed “sea snail” cell clusters with one blunt end and one tip end (Figure 3(a)). H&E staining was used to demonstrate the histological features of the cell clusters in the upper chambers. In the EPP group, cell clusters were composed of 7-10 cells with glandular structure. In the dorsal skin group, nonglandular cell clusters consisted of 4-5 cells. The “sea snail” structure demonstrated polarity of one blunt end and one tip end, with distinct mitosis. In the blank control group, cell clusters were made of only 2-4 cells (Figure 3(b)). Immunofluorescence staining was used to identify cellular phenotypes and specific markers of the cell clusters (Figure 3(c)). The glandular cell clusters in the EPP group showed K5 and K14 positive, which are the specific markers of epithelial stem cells. K8 and K18 were expressed in some clusters with ductal structure. And K8 and K18 belong to the intermediate filament family; they are specifically expressed in sweat gland cells in epithelia [2]. It indicated that the glandular cell clusters may be the developing sweat gland cells. In the dorsal skin group, K5, K14, and K8 were expressed in the nonglandular cell clusters, but K18 was negative in this group. It indicated that the “sea snail” cell clusters did not involve in sweat gland development. In the blank control group, cell clusters only expressed K5 and K14. K8 and K18 showed negative in these clusters.

### 3.3. Detection of Key Soluble Factors in the Model of Sweat Gland Morphogenesis

**Figure 4:**
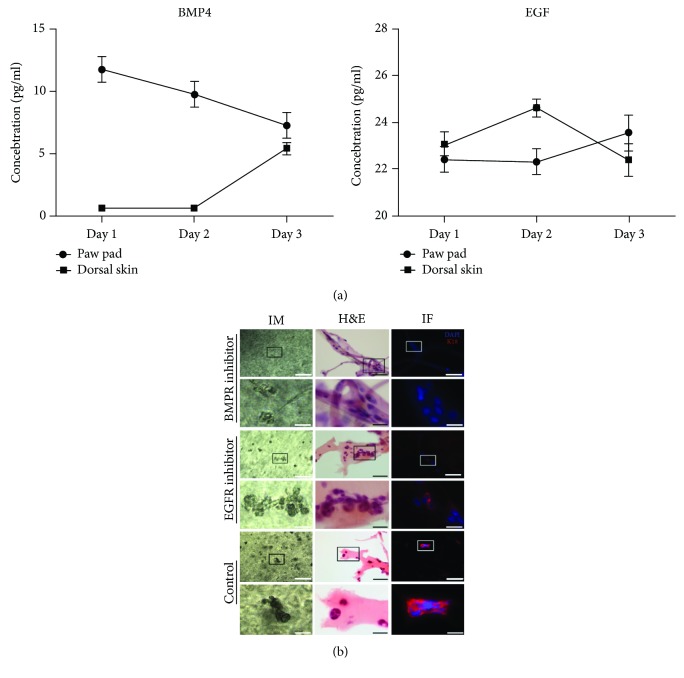
Detection of key soluble morphogens in the system of sweat gland morphogenesis and the impact of BMP receptor inhibitor and EGF receptor inhibitor on sweat gland development in vitro. BMP4 and EGF demonstrated sharped difference in the medium of the sweat gland organogenesis system. BMP receptor inhibitor could block the formation of sweat gland in this system, while EGF receptor inhibitor significantly reduced the expression of K18. (a) The variation tendency of BMP4 and EGF in the medium of system. The error bar meant the standard error of BMP4 and EGF concentrations in different systems at different time points. (b) The impact of BMP receptor inhibitor and EFG receptor inhibitor on sweat gland morphogenesis. In comparison with the control group, EGF receptor inhibitor significantly reduced the expression of K18, but glandular structure was still observed. BMP receptor inhibitor completely blocks the expression of K18, and no glandular structure was observed. All the nuclei were counterstained with DAPI (DAPI: blue; K18: red; bars = 200 *μ*m and 50 *μ*m; K18: cytokeratin 18; IM: light microscope; H&E: hematoxylin-eosin staining; IF: immunofluorescence staining).

In order to test the dynamic changes of key soluble morphogens in this model, we detected the concentration of BMP4 and EGF in the medium of this coculturing model. The concentration of BMP4 was decreasing with time in the supernatant of the EPP group. In the dorsal skin group, BMP4 was increasing along with time. In the test system, BMP4 in the EPP group was higher than that in the dorsal skin group at days 1 and 2 (*p* < 0.05). But there is no significant statistical difference of BMP4 concentration between two groups at day 3 (*p* > 0.05). Otherwise, the concentration of EGF was increasing along with time in the supernatant of the EPP group. In the dorsal skin group, EGF increased at first and then decreased in the supernatant. EGF in the EPP group was higher than that in the dorsal skin group at day 2 (*p* < 0.05), but there is no significant statistical difference of EGF level between two groups at days 1 and 3 (*p* > 0.05). All data are shown in Figure 4(a).

In order to validate the effect of BMP4 and EGF in sweat gland morphogenesis in the test system, BMP4 receptor inhibitor and EGF receptor inhibitor were added into the system, respectively. When BMP4 signal was blocked, no glandular cell clusters were found by inverted microscopy or H&E staining. And there was no cell clusters with K18 positive in immunofluorescence staining. While EGF was blocked, glandular cell clusters could still be found by inverted microscopy and H&E staining. But K18 expression was significantly reduced in these cell clusters (Figure 4(b)).

## 4. Discussion

During the development of sweat glands, the epithelial-mesenchymal crosstalk is mastering and regulating the process. All eccrine sweat gland cells are derived from epithelial stem cells or epithelial progenitor cells in mammals [1, 2]. In mice, the eccrine sweat glands specifically reside in the paw pad. In the sweat gland niches, signals between cells and matrix have determined the final cellular fate of epithelial stem cells. In addition to the structural component of extracellular matrix, secreted soluble factors are bridging the information between cell-to-cell and cell-to-matrix. Due to limited interacting surface between epithelia and underlying mesenchyme, secreted soluble factors may lead the process.

Morphogens, growth factors, or other soluble regulatory factors could activate or stimulate crucial signal pathways involving development and organogenesis. EGF could induce bone marrow-derived mesenchymal stem cells to form sweat gland tissue with more than 2 weeks of in vitro culture. It is also reported that umbilical cord mesenchymal stem cells could be induced into sweat gland cells with the impact of keratinocyte growth factor [10, 11]. Studies have demonstrated that many soluble factors are involved in the embryonic morphogenesis of sweat glands, including EGF, FGF, FoxA1, BMP4, and EDA. Besides, the concentration of these factors is not maintained at a certain level throughout the process [11–13]. In this study, the authors found BMP4 released by EPP tissue was gradually decreasing, and EGF from EPP was gradually increasing. Nevertheless, BMP4 released from EPP was constantly higher than that from dorsal skin within three days of in vitro culture. This phenomenon implied that multiple factors might exert coordinating functions in sweat gland morphogenesis. Using a single factor with stable concentration could be one reason of low efficiency in typical approaches for inducing sweat glands. In order to find an effective way of induction, this study directly used EPP tissue to induce ESCs into sweat glands in a 3D condition.

In this test system, key soluble factors were released from embryonic tissue. Whether sweat glands could develop normally in vitro is the first question that should be answered. Previous studies have adopted organotypic culture of embryonic tissue as a model of development [12–14]. In this research, paw pad tissue at embryonic stage of 15.5 day was cultured in vitro. After one day of culture, placode structure was observed at the basal layer of epithelia. Epithelial bud and glandular structure were successively observed at two and three days of culture. Specific markers, K14 and K5, were expressed and distributed in the placode, bud, and glandular structure of organotypic tissue as well as their patterning in paw pad at embryonic stage of 15.5 to 18.5 days. The findings indicated that sweat gland development in vitro for 3 days could keep pace with physiological morphogenesis by and large. After 3 days, epithelial and mesenchymal tissues exhibited irregular structure which the histological patterning was hardly recognizable. Sweat glands start to develop at 16.5 day in embryonic mice, and its duct part is present at 18.5 day at embryonic stage. It implies that 16.5-18.5 days at embryonic stage of mice is the most crucial time for sweat gland development, and latest research also found that it was the “window time” which cellular fate of skin appendage could be switched by appropriate stimulation [15]. Therefore, EPP tissue could be served as a “mini factory” for releasing key soluble factors of sweat gland development, in order to precisely simulate the physiological sweat gland development in vitro.

Induction of stem cells with the aid of tissue-specific extracellular matrix, a ubiquitous method in the field of regenerative medicine, could give rise to directional differentiation [16]. Our previous research has demonstrated that ESCs could successfully differentiate into sweat glands with EPP homogenate in extracellular matrix. It indicated that some deterministic molecules of cellular fate and differentiation must have existed in the EPP homogenate. In this experiment, glandular cell clusters were formed in the inducing system with EPP tissue in the lower chamber. Besides, these clusters also expressed sweat gland specific markers, K8 and K18. Meanwhile, “sea snail” clusters were observed with dorsal skin in the lower chamber. And the nonglandular cell cluster only showed K8 positive. It is believed that K8 is also expressed in hair follicular cells, sebaceous gland cells, and sweat gland cells in the integumentary system. While this study did have some limitations, as the embryonic tissue could only maintain its structure and function for 3 days in vitro, it has restrained the research period of the test system.

The test system in this experiment has paved the way for investigating soluble factors in sweat gland organogenesis, as Transwell plate could prevent the direct contact of EPP tissue and ECSs. Our results showed that BMP4 receptor inhibitor could hinder the formation of glandular cell clusters, but EGF receptor inhibitor did not prevent the process. It may imply that BMP4 has indispensable functions in sweat gland development, while EGF may not be the irreplaceable factor in this period of development. In line with this approach, our 3D models could replace animal modeling as an alternative bioactive screening model. In addition, these functional 3D models could also be implemented into a full skin model missing skin appendages like sweat glands for studying wound healing processes.

## Data Availability

All data are fully available without restriction. All data were included in the paper.
